# Ogeechee Medical Association

**Published:** 1887-03

**Authors:** 


### OGEECHEE MEDICAL ASSOCIATION.

At a meeting, for electing officers for the year 1887, of the Ogeechee Medical Association, which is composed of physicians of the counties of Burke, Screven. Bulloch, and Emanuel, held bi-monthly at Millen, Ga., the following were chosen: Dr. E. W. Lane, President; Dr. E. A. Perkins, President for Burke Co.; Dr. Alexander Matthews, President for Screven Co.; Dr. John I. Lane, President for Bulloch Co.; Dr. D. E. Gay, President for Emanuel Co.; Dr. R. Y. Lane, Secretary and Treasurer; Dr. B. L. Clifton, Assistant Secretary.

We congratulate the brethren upon the organization of this new medical association, and trust it will be a prosperous one.

